# Membrane-free culture and real-time barrier integrity assessment of perfused intestinal epithelium tubes

**DOI:** 10.1038/s41467-017-00259-3

**Published:** 2017-08-15

**Authors:** Sebastiaan J. Trietsch, Elena Naumovska, Dorota Kurek, Meily C. Setyawati, Marianne K. Vormann, Karlijn J. Wilschut, Henriëtte L. Lanz, Arnaud Nicolas, Chee Ping Ng, Jos Joore, Stefan Kustermann, Adrian Roth, Thomas Hankemeier, Annie Moisan, Paul Vulto

**Affiliations:** 1 Mimetas BV, JH Oortweg 19, 2333CH Leiden, The Netherlands; 2 Roche Innovation Center Basel, F. Hoffmann-La Roche Ltd, Grenzacherstrasse 124, 4070 Basel, Switzerland; 3 Leiden Academic Centre for Drug Research, Leiden University, Einsteinweg 55, Leiden, 2333CC The Netherlands

## Abstract

In vitro models that better reflect in vivo epithelial barrier (patho-)physiology are urgently required to predict adverse drug effects. Here we introduce extracellular matrix-supported intestinal tubules in perfused microfluidic devices, exhibiting tissue polarization and transporter expression. Forty leak-tight tubules are cultured in parallel on a single plate and their response to pharmacological stimuli is recorded over 125 h using automated imaging techniques. A study comprising 357 gut tubes is performed, of which 93% are leak tight before exposure. EC_50_-time curves could be extracted that provide insight into both concentration and exposure time response. Full compatibility with standard equipment and user-friendly operation make this Organ-on-a-Chip platform readily applicable in routine laboratories.

## Abstract (web summary)

Efforts to determine the effects of drugs on epithelial barriers could benefit from better in vitro models. Here the authors develop a microfluidic device supporting the growth and function of extracellular matrix-supported intestinal tubules, and evaluate the effect of staurosporine and acetylsalicylic acid on barrier integrity.

## Introduction

Dysfunction of epithelial barriers as a result of pathological states or drug-induced toxicity can lead to life-threatening conditions and halt drug development at all clinical stages. Epithelial barrier disruption is mainly manifested by an increased para-cellular permeability of the epithelium. In vitro testing of para-cellular permeability of epithelial barriers is most commonly achieved by cultivating cells on a rigid membrane that separates two medium-containing chambers under static conditions. Such conventional Transwell systems are poorly suited for high-resolution kinetic measurements and image-based readouts, and therefore provide only limited information on the underlying mechanisms, leading to barrier disruption. More importantly, it does not comply with the current paradigm in cell culture that is steadily shifting towards three-dimensional cultures, extracellular matrix (ECM) embedment and addition of perfusion flow^1–5^.

The field of microfluidics has rapidly gained momentum in the realm of in vitro modeling^3^. Inherent to its dimensions, microfluidic techniques are uniquely suitable to connect with epithelia of tubular shapes in order to provide shear stress and continuous medium refreshment through perfusion. Typical microfluidic solutions make use of artificial membranes to enable apical-basal access to the epithelia^6^, thus not accommodating an ECM that is a crucial parameter in cell signaling involved in differentiation and epithelial-to-mesenchymal transition. Also, microfluidic techniques, which are typically presented as single chips^7^, need to be parallelized in order to deliver readouts for multiple compounds, dilutions, replicates and controls. User-friendly operation and compatibility with state-of-the-art readouts, such as high content imaging (HCI)-based multiplexed cellular and molecular analyses, are crucial prerequisites for perfused, ECM-embedded cell culture techniques to become a new standard.^8^

We developed a methodology to culture perfused, ECM-supported epithelia and interrogate their barrier function in a membrane-free manner. As an example, we developed a model of intestinal tract epithelium that exhibits cellular polarization, tight junction formation, and expression of key receptors. Forty gut models were grown in a tubular shape in the OrganoPlate platform that was accessible from both the apical and basal sides. The tubes were assessed for barrier integrity and exposed to staurosporine and acetylsalicylic acid (aspirin) for 125 h. From 330 tubes used in these experiments, 93% were leak tight before exposure. The experiment was repeated using real-time parallel time-lapse imaging, in which tubes were stable up till 6 to 8 h. EC_50_-time curves provide insight in concentration response at increasing exposure time in one single experimental run.

## Results

### Intestinal tube culture in OrganoPlate

**Fig. 1:**
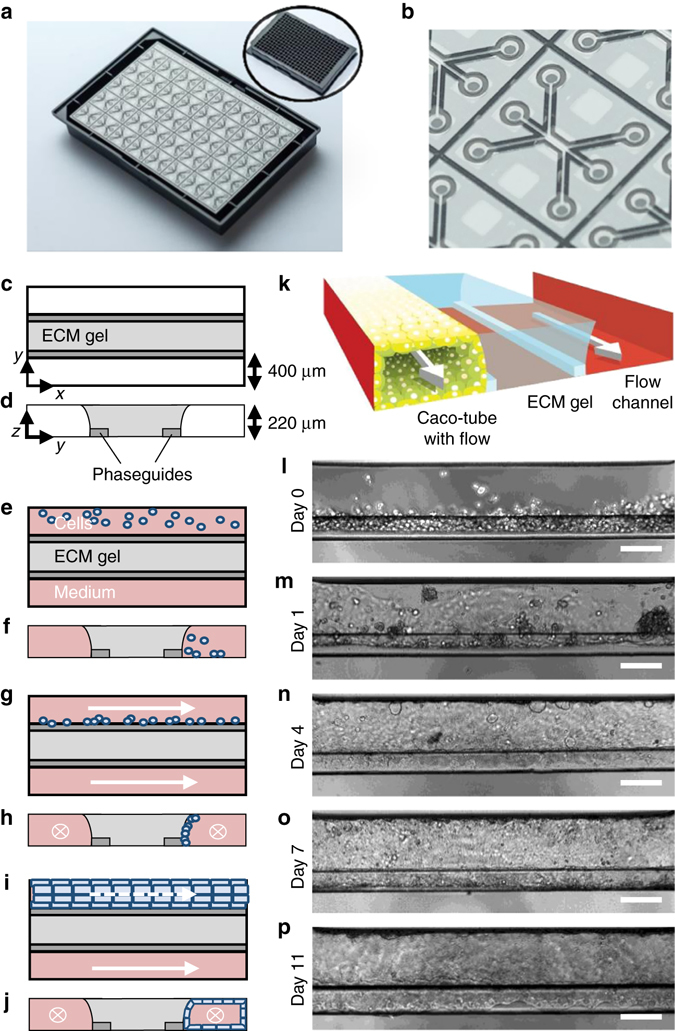
Overview of the method for modeling intestinal tubules in the OrganoPlate platform. **a** Photograph of the bottom of an OrganoPlate showing 40 microfluidic channel networks with inlay showing the top view of the 384-well plate format device; **b** Zoom-in on a single microfluidic channel network comprising three channels that join in the center. **c**, **e**, **g**, **i** Horizontal projection and **d**, **f**, **h**, **j** vertical cross section of center region for subsequent steps in establishing the gut model. **c**, **d** An extracellular matrix gel (*light gray*) is patterned by two phaseguides (*dark gray*), **e**, **f** culture medium is introduced in the two lanes adjacent to the ECM gel, one of which comprises cells. **g**, **h** Cells are allowed to settle against the ECM gel surface by placing the plate on its side. **i**, **j** Upon application of flow, cells form a confluent layer lining the channel and gel surfaces, resulting in a tubular shape. **k** 3D artist impression of the center of a chip comprising a tubule, an extra cellular matrix gel and a perfusion lane; two phaseguides (*white bars*) are present that define the three distinct lanes in the central channel. The tubule has a lumen at its apical side that is perfused. **l**–**p** Phase-contrast images of the formation of the tubular structure at day 0, 1, 4, 7, and 11, respectively. *Scale bars* are 100 µm

Figure 1 shows the OrganoPlate platform, which encompasses 40 microfluidic cell culture structures embedded in a standard 384-well microtiter plate format (Fig. 1a, b)^9, 10^. Each microfluidic channel structure is comprised of three lanes that are connected to corresponding wells of a microtiter plate that function as inlets and outlets to access the microfluidic culture. The lanes join in the centre of the structure where two capillary pressure barriers are present called phaseguides^11^. Figure 1c–j shows a schematic representation of vertical and horizontal cross-sections of the centre of a microfluidic structure and the method of growing a tubular structure. First, an ECM gel is introduced in the central lane (Fig. 1c, d). The phaseguides are used to selectively pattern the ECM gel in the central lane by meniscus pinning. The meniscus stretches beyond the phaseguide, leading to a curved shape. After ECM gelation, epithelial cells are seeded in one lateral lane, allowing them to sediment directly against the ECM gel by placing the titre plate in a vertical position, i.e., standing on one side **(**Fig. 1e–h **)**. Upon attachment of the cells, the plate is horizontally placed on an interval rocker that induces flow by reciprocal leveling between reservoirs **(**Supplementary Fig. 1). Upon application of flow, cells proliferate and start lining all surfaces of the perfusion channel, forming a confluent tubular structure (Fig. 1i, j). The tubules have a lumen that is connected to the in- and outlet of the respective lanes, making then accessible for perfusion with medium and for apical compound exposure. The basal side of the epithelium is facing the ECM gel and can be accessed by the second perfusion lane on the opposite side of the ECM gel lane. Figure 1k depicts an artist impression of the 3D configuration of the tube, showing that the tube is grown directly against the ECM, without the presence of artificial membranes **(**Fig. 1k **)**.

For modeling of the intestinal barrier, the human intestinal colorectal adenocarcinoma cell line (Caco-2) was used. Figure 1l–p shows phase-contrast pictures of tube formation at day 0, 1, 4, 7, and 11, respectively. On day 0, cells are seeded against the ECM and start colonizing the glass walls to form a confluent tube (Fig. 1n–p). Perfusion was crucial for tube formation. Tubes were formed in 3 days and optimal barrier function was found at day 4 **(**Supplementary Fig. 1 **)**.

### Differentiation and polarization marker expression

**Fig. 2:**
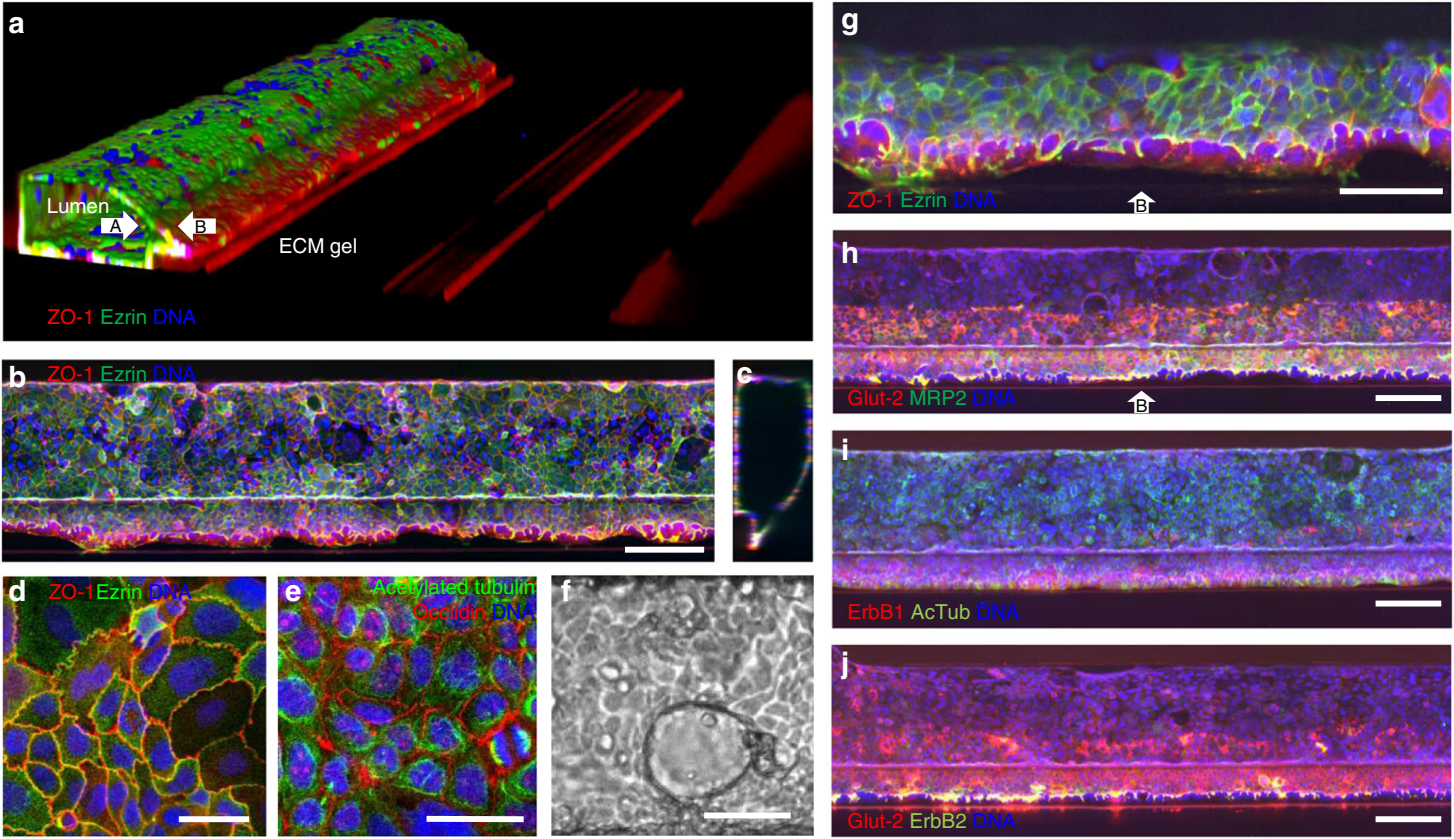
Tubule characterization by immunofluorescent staining. **a** 3D reconstruction of a confocal *z*-stack showing tubular morphology with a lumen. *White arrows* indicate the apical (*A*) and basal (*B*) sides. The tube is stained for tight junctions (ZO-1 in *red*) and brush borders (ezrin in *green*). **b** Max projection and **c** vertical cross-section of the tubular structure in **a**; **d**, **e** zoom of the epithelial layer at the bottom of the tube exhibiting **d** tight junctions (ZO-1 in *red*) and brush borders (ezrin in *green*), and **e** acetylated tubulin (*green*) and occluding (*red*). **f** Phase-contrast image showing dome formation. **g** Zoom of a *z*-slice of the tube in **a** of the cell layer on top of the phaseguide showing apical positioning of ezrin, indicating polarization of the tube (*white arrow* indicates basal side B). **h** Expression of glucose and MRP2 transporters, respectively stained with Glut-2 in *red* and MRP2 stain in *green*. Both Glut-2 and MRP2 show significantly higher signal against the collagen gel compared to the regions that are not exposed to the collagen, indicating increased expression levels. Both stains clearly stain the apical side of the tube. For *z*-slices above the phaseguide at a higher magnification see Supplementary Fig. 2b. **i** ErbB1 (*red*) and acetylated tubulin (*green*) expression. ErbB1 expression levels appear higher against the collagen. **j** Co-staining of Glut-2 transporter and ErbB2 receptor; both stains show higher signal levels against the collagen gel. ErbB2 is primarily expressed pericellularly (see also Supplementary Fig. 2d for a zoom)). All tubes are fixed after 4 days in culture. Nuclei are stained *blue* with Draq5 (**a**–**c**, **g**–**j**) and DAPI (**d**, **e**). *Scale bars* in *white* are 100 µm with the exception of **d**, **e**, **f**, and **g**, where they are 50 µm. *Z*-slices just above the phaseguide at higher magnification of the images **g**–**j** are available in Supplementary Fig. 2. All images are representative of at least three biological and at least three technical replicates

Figure 2a shows a 3D reconstruction of confocal fluorescence micrographs of the gut tube. The tube has a clear lumen and lines the perimeter of the gel and perfusion lane. Caco-2 cells in the confluent tubule display tight junctions and brush border formation as shown by immunofluorescence staining of ZO-1 and ezrin, respectively^12^ (Fig. 2a–d, g). Figure 2e shows localization of acetylated tubulin (microtubules) and occludin (tight junctions)^13^. Dome-formation is observed, indicative of active fluid transport and intact epithelial barrier function^14, 15^ (Fig. 2f **)**. Figure 2h–j shows maximum intensity projection images of tubes stained for Glut-2, MRP2, ErbB1, and ErBb2. Cells in contact with the ECM showed a strongly increased expression of the transporters Glut-2, MRP2 and to a lesser extend ErbB1 and ErbB2 receptors.^16^ These staining results illustrate the crucially instructive role that the ECM plays in cellular differentiation and protein expression. Furthermore, characteristics of the ECM gel surface, such as its (bio-)chemical composition and mechanical characteristics, allow the formation of tissue structures observed in vivo.^17^ Polarization of the cell layer against the gel is best visualized at the contact line between the gel meniscus and the phaseguide, at the bending point of the cell layer where apical-basal polarization is in the horizontal plane. This is the most right-hand part of the tube in Fig. 2a or the bottom side of the tube in Fig. 2b. Figure 2g and Supplementary Fig. 2 show single *z*- slices at this bending point, just above the phaseguide. Tubes are the same as the images of Fig. 2b, h–j, but depicted as single *z*-slices and at higher magnification. Polarization is confirmed by localization of brush borders (ezrin) and the MRP2 transporter on the apical side as shown in Fig. 2g, and Supplementary Fig. 2a, b, while ErBb2 is positioned pericellularly (see Supplementary Fig. 2d). At least 10 Caco tubes were stained with each marker that were grown on at least four different days for at least four different passage numbers of cells prior to seeding. Figures show a representative selection of results.

### Barrier integrity

**Fig. 3:**
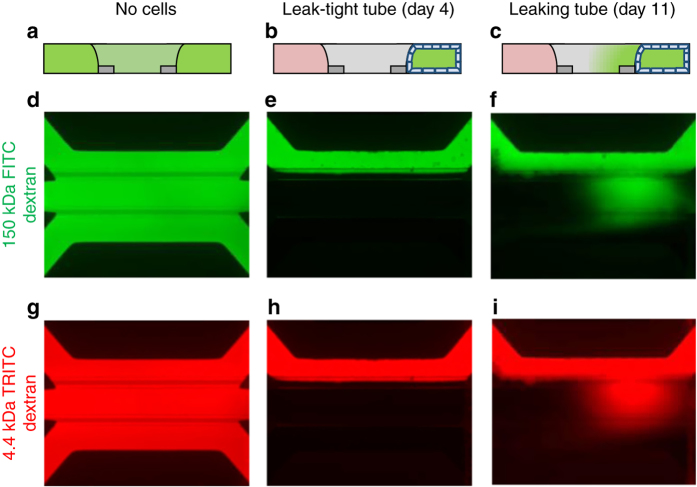
Barrier integrity assay in OrganoPlate. A fluorescent dye is inserted in the channel comprising the tube. Integrity of the tube barrier is quantified by measuring the amount of dye that is leaking out of the tube into the adjacent gel channel. **a**–**c** Sketch in vertical cross section showing fluorescence distribution: **a** in absence of a tube, **b** for the case of a leak-tight tube and **c** for a leaky tube. **d**–**i** Fluorescent images of microfluidic chips perfused with fluorescent molecules show experimental results for: gel only (**d**, **g**), leak-tight tube (**e**, **h**), and leaky tube (**f**–**i**) using both 150 kDa FITC-dextran and 4.4 kDa TRITC-Dextran during the same experiment

Barrier function of the Caco-2 tubes was assessed by perfusion with a fluorescent probe in culture medium through the tube lumen, followed by the determination of fluorescence levels in the basal gel region, normalized to the fluorescence in the lumen. This is illustrated in Fig. 3a–i. Both a high molecular weight fluorescent probe (150 kDa FITC-dextran) and a lower molecular weight probe (4.4 kDa TRITC-dextran) were added to the medium that is perfused through the lumen of the tube. In absence of an intact tubular structure, the fluorescent probes leak into the gel and the basal side perfusion channel (Fig. 3a, d, g), while for a fully intact barrier, the fluorescent probes are retained in the lumen of the tube (Fig. 3b, e, h). Upon (partial) loss of barrier function, e.g., through drug-induced toxicity, the fluorescent probe leaks out of the lumen towards the basal side, yielding a higher signal in the ECM (Fig. 3c, f, i). Barrier integrity was measured using a HCI system, allowing monitoring of 40 tubes in parallel. To quantify the integrity of the barrier, the fluorescence level was measured in the gel region and normalized to the fluorescence level in the luminal side to compensate for bleaching effects. Upon reaching a fluorescence value of 0.4, barrier integrity of a tube was considered lost. The barrier integrity of 24 tubes was tracked on day 4, 7 and 11 of culture. As depicted in Supplementary Fig. 1c, it was found that, at day 4, all tubes were leak tight, while at day 7 and 11, three and seven tubes were leaky, respectively. Therefore, barrier integrity measurements are performed at 4 days of culture.

### Drug-induced barrier disruption

**Fig. 4:**
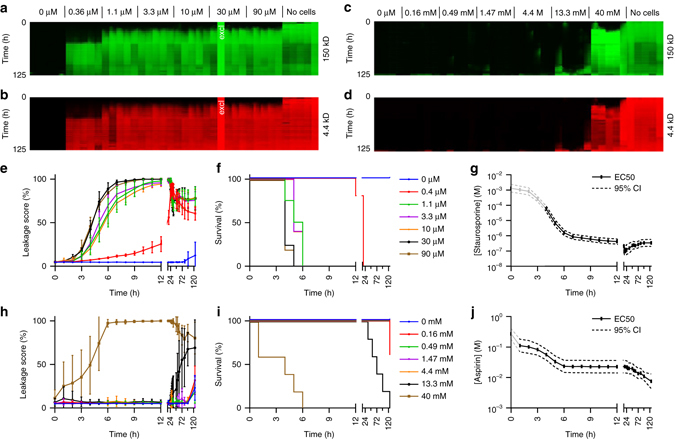
Drug-induced loss of barrier integrity is observed over time in a concentration-dependent manner. Results shown for staurosporine (**a**, **b**, **e**–**g**) and aspirin (**c**, **d**, **h**–**j**). **a**–**d** Array of fluorescence micrographs of the gel region showing distribution of the 150 kDa FITC-Dextran (**a**, **c**), and 4.4 kDa TRITC-Dextran (**b**, **d**) over time and for various compound concentrations; the loss of barrier integrity results in an increased fluorescent signal. Measurements are taken at 1-h intervals up to 12 h, at 16 h, from 24 to 36 h at 1 h interval, and at 48, 53, 60, 72, 82, 96, and 125 h. In between each interval, the OrganoPlate was placed back into the incubator on the interval rocker platform to maintain the perfusion flow. Five technical replicates of each concentration of a compound were measured on a single plate. One well was excluded from further data analysis, because of a pipetting error (marked with “excl” in *white*). **e**, **h** The progression of the loss of barrier function over time is plotted as the ratio between fluorescent signal in apical and basal regions for the various concentrations of staurosporine (**e**) and aspirin (**h**), where the plotted *line* is the mean of five replicate exposures and *error bars* depict the standard deviation. **f**, **i** Kaplan–Meier curves were generated where survival was defined as showing a leakage score below 40%. Overlapping curves were shifted by 1% for clarity purposes. **g**, **j** EC_50_ values are plotted as a function of exposure time. EC_50_ values were obtained by fitting a concentration-response curve at each time point based on non-linear regression of leakage scores using normalized response and standard slope and were plotted including 95% confidence interval (*CI*). EC_50_ values obtained from time points before the first event in the Kaplan–Meier plot, as indicated by a grayed out line, should be interpreted with caution as the curve fit could be dominated by noise rather than biological effect. All shown graphs were derived from data acquired using 150 kDa FITC dextran. Technical replicates are defined as tubes seeded on the same plate and exposed in the same experimental session. Independent full replicate series were run for both staurosporine and aspirin that are displayed in Supplementary Figs. 3 and 4

Barrier integrity of 4-day-old Caco-2 tubes was assessed during a 125-h apical exposure to various concentrations of staurosporine (0.4–90 μM), an inducer of apoptosis,^18^ and aspirin which affects tight junctions^19^ (0.16–40 mM). Fluorescence levels were measured at 1-h intervals from 1 to 12 h, and 24 to 36 h, as well as at 16, 48, 53, 60, 72, 82, 96, and 125 h. Between measurements, the OrganoPlate was placed back on the rocker platform to maintain flow. Figure 4a–d depicts arrays of images showing the fluorescence in the gel at each time-point for both FITC- and TRITC-dextran. Measurements for each compound were taken on a single OrganoPlate with five replicates per concentration. The staurosporine and aspirin studies were executed five and three times, respectively in separate experimental sessions (see Supplementary Fig. 4).

Fluorescence images of one single OrganoPlate depicted in the arrays of Fig. 4a–d and their quantification as depicted in Fig. 4e, h show that barrier integrity gradually diminished over time for all concentrations of staurosporine and for the two highest concentrations of aspirin. Results can also be visualised by generating Kaplan–Meier plots for loss of barrier integrity, in which events are defined as the fluorescence ratio reaching 40% (Fig. 4f, i). This approach is particularly useful for less potent toxicants, where EC_50_ determination suffers from a lack of data of high effect. Aspirin, which has a different mode of action, involving tight junction disruption and proliferation inhibition, instead of apoptosis induction^20, 21^, causes much less barrier disruption at relevant concentration. The Kaplan–Meier curve, however, does show a highly significant trend of loss-of-barrier function at higher concentration (*P* < 0.0001 for both curve difference and trend significance, derived from log-rank tests) (Fig. 4i). An EC_50_ value was estimated for each time point by fitting the concentration-response curve based on non-linear regression of the logarithm of the compound concentration vs. the normalized fluorescence, assuming a top and bottom plateau at 0 and 100% fluorescence. With increasing exposure times, a shift of EC_50_ toward lower compound concentrations was observed (Fig. 4g, j). The 95% confidence interval of the extracted EC_50_ values indicates the robust data over the entire exposure time. EC_50_ values extracted at time points before the first event in the Kaplan–Meier plots should be interpreted with caution, as baseline fluorescence is likely to dominate the curve fitting rather than a biologically relevant signal.

Supplementary Fig. 3 shows an independent repeat of the study in Fig. 4 using cells seeded at different passage numbers in separate experimental sessions. Supplementary Fig. 3h–j, n–p also shows the data analysis for 4.4 kDa TRITC-dextran.

Supplementary Fig. 4 shows an overlay of the EC_50_ curves for 5 independent experimental series of staurosporine (Supplementary Fig. 4a, b) and aspirin (Supplementary Fig. 4c, d), based on both 150 kDa FITC-dextran (Supplementary Fig. 4a, c) and 4.4 kDa TRITC-dextran (Supplementary Fig. 4b, d) analysis. Independent experimental series were performed using cells at different passage numbers, separate plates, and separate experimental sessions. The high degree of similarity between the EC_50_ time curves for the full replicate series are a powerful illustration of the robustness of the method.

In parallel to fluorescence images, phase-contrast pictures were taken at selected time points. An example of tube morphology as a response to 96 h of staurosporine exposure is included in the Supplementary Information (Supplementary Fig. 5). Tubes are fully deteriorated for the highest concentration staurosporine and damages can be observed for exposure to 30 µM staurosporine. For lower concentrations, tubes appear intact, while fluorescence images show that barrier integrity is lost. This indicates that loss of barrier function at higher concentrations of staurosporine is primarily due to cell death. Dead cells are flushed away by the perfusion flow.

For comparison, a similar experiment in a conventional Transwell was performed, which revealed a lower sensitivity for barrier disruption compared to the OrganoPlate, showing no significant difference between controls and staurosporine exposed wells after 4 h (Supplementary Fig. 6), while a clear effect is already apparent in the OrganoPlate results. In addition to improved morphological maturity of the 3D-perfused culture, the increased sensitivity of the model can be attributed to a decreased dead volume and higher surface-to-volume ratio of the microfluidic system as compared to Transwell systems. In a Transwell, the FITC-dextran was strongly diluted in the large target volume when crossing the barrier. By contrast, in OrganoPlates, the fluorescence is measured directly in the ECM after crossing the epithelial membrane. Since no dilution step is involved here, a much higher signal-to-noise ratio is obtained. Sensitivity of the microfluidic assay is such that it can be used as a binary assay, in which the exposure time at which leakage is observed is indicative of the toxicity of the compound.

### Real-time measurement

**Fig. 5:**
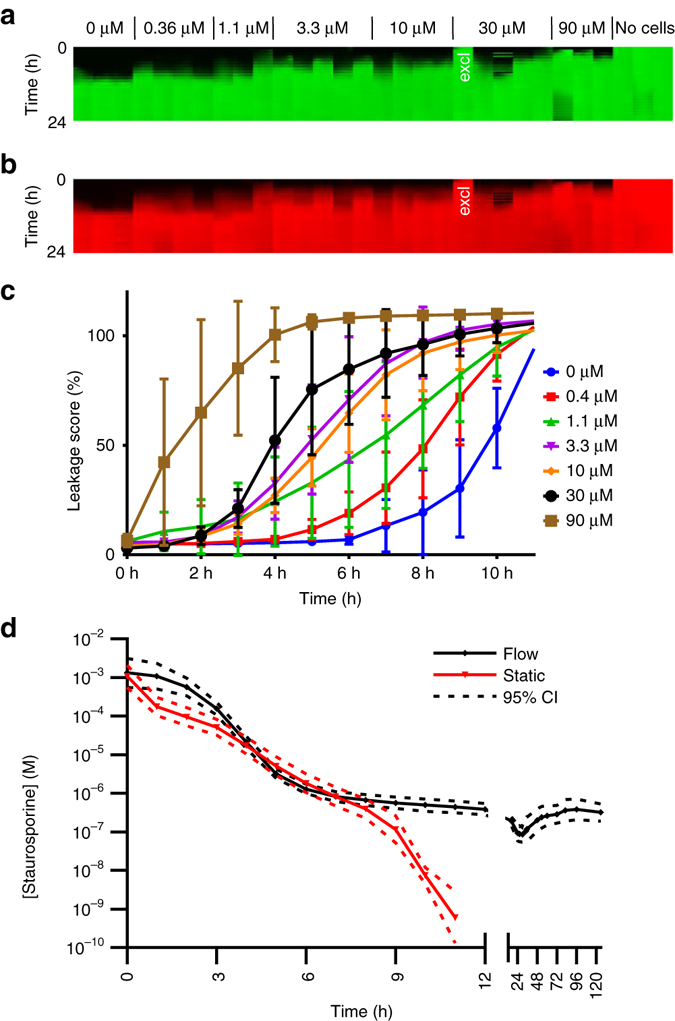
Drug-induced loss of barrier integrity as a function of staurosporine concentration measured in real-time. **a**, **b** Array of fluorescence micrographs of the gel region showing distribution of the 150 kDa FITC-Dextran (**a**) and 4.4 kDa TRITC-Dextran (**b**) over time and as a function of compound concentration; the OrganoPlate was continuously kept in an incubated automated microscope. Pictures were taken at 1 h intervals. One data point was excluded for the fact that the tube appeared leaky at the first time point (marked with “excl” in *white*). **c** The progression of the loss of barrier function over time shows that untreated controls lose barrier integrity at 6–8 h due to lack of flow. The plotted *line* is the mean of 3–5 technical replicate exposures and *error bars* depict the standard deviation. **d** EC_50_ values over time for real-time measurement without flow and for measurement in intervals with flow induced between measurement (overlay with the graph of Fig. 4g). EC_50_ values with and without flow are similar for the initial 8 h of measurement

Figure 5 shows another concentration response experiment with staurosporine, but this time the OrganoPlate was continuously kept inside the microscope throughout the experiment. A conditioned high content imager was used to maintain appropriate CO_2_, temperature and humidity. Flow was absent in this experiment, as the high content imager did not provide rocking. As can be observed in fluorescent images and quantification thereof, vehicle control tubes started leaking after 6 to 8 h of imaging. This can most likely be attributed to suboptimal conditions, including a lack of perfusion flow. Nevertheless, a clear concentration-response effect could be observed. An EC_50_-time curve was extracted and overlaid in red with the curve from the experiments with rocking in black (as shown previously in Fig. 4g), showing similar curves for up to 8 h **(**Fig. 5c **)**. The advantage of incubation in the microscope is that a higher time resolution can be obtained.

In the experimental series of Figs. 4, 5, Supplementary Fig. 3 and Supplementary Fig. 4 10 OrganoPlates were used, comprising a total of 357 gut tubes and 33 ECM-only negative controls in total. Two tubes were excluded because of pipetting errors and 26 because of insufficient barrier function after 1 h, yielding 93% of leak-tight tubes at the onset of drug exposure.

## Discussion

In summary, we present a unique methodology for assessing the barrier integrity of 40 leak-tight, polarized epithelial gut tubes in parallel using HCI. It is for the first time that a comprehensive method is presented to interrogate perfused epithelia tubules that are exposed to an ECM. The system allows sensitive, real-time interrogation of compound effects on barrier integrity, yielding insight in both exposure concentration and exposure time effects. The method has been robustly demonstrated for over 350 gut tubes and over 20,000 datapoints, making this to our knowledge the largest published Organ-on-a-Chip data set so far. The method can be applied to other epithelia as well as translated to disease models. The co-culture capabilities of the platform^9^ can be explored to create complex tissue configurations, for example, by incorporating mesenchymal and immune cells in the ECM adjacent to the epithelial tubes. The system outperforms classical techniques such as Transwell systems in terms of sensitivity, ease of use and (multiplexed) readout flexibility, as well as reagent, cell and time consumption. More importantly, it allows for the first time non-expert end-users to adopt Organ-on-a-Chip technology in their laboratories, without need for specific microfluidic skills or dedicated equipment.

## Methods

### Cell culture

The human colon adenocarcinoma cell line Caco-2 (86010202, Sigma-Aldrich) was cultured on T75 flasks in EMEM (No. 30-2003, ATCC), 10% FBS (No. F4135, Sigma), 1% NEAA (No. 11140-050, Life Technologies) and 1% penicillin/streptomycin (Sigma #P4333). Caco-2 cells between passage 45 and 60 were used for all experiments. Cells were routinely tested for mycoplasma contamination and found negative.

### OrganoPlate culture

OrganoPlate culture was performed using three-lane OrganoPlates with 400 µm × 220 µm (w x h) channels (Mimetas BV, the Netherlands). Phaseguides had dimensions of 100 µm × 55 µm (w × h). Gel and perfusion channels have a length of 9 mm and 13 mm, respectively. 2 µl of gel composed of 4 mg/ml Collagen I (AMSbio Cultrex 3D Collagen I Rat Tail, 5 mg/ml, Cat. 3447-020-01), 100 mM HEPES (Life Technologies, 15630–122) and 3.7 mg/ml NaHCO_3_ (Sigma, Cat. S5761) was dispensed in the gel inlet and incubated 30–45 min at 37 °C. Caco-2 cells were trypsinized using 0.5% trypsin in PBS/EDTA (Sigma, T3924), aliquoted and pelleted (5 min, 100 × g). The cells were applied to the system by seeding 2 µl of 1 × 10^7^ of cells/ml in the outlet of the top medium channel. Subsequently, the OrganoPlate was put on the side for 20 min to allow the cells to sediment against the ECM. This was followed by addition of 50 µl medium to the outlet of the top medium channel and the OrganoPlate was again incubated on the side for 3–4 h at 37 °C to complete cell attachment. After incubation, medium was added up to a total of 50 µl on both inlets and both outlets. The OrganoPlate was placed horizontally in the incubator (37 °C 5% CO_2_) on an interval rocker switching between a + 7° and −7° inclination every 8 min (Mimetas Rocker Mini), allowing bi-directional flow. Medium (50 µl each on inlet and outlet) was refreshed every 2–3 days.

### Transwell culture

Caco-2 cells (60 × 10^3^ cells per cm^2^) were seeded on Transwell inserts (24-well, Transwell, Costar #3470-Clear, 0.4 µM pore size) and cultured for 21 days in EMEM supplemented with 10% fetal calf serum (FCS), and penicillin/streptomycin (Sigma #P4333). Medium was refreshed every 2–3 days, both 100 µl on apical (insert) and 500 µl on basal side of the Transwell.

### Immunohistochemistry

Caco-2 tubules were fixed with 3.7% formaldehyde (Sigma No. 252549) in PBS (phosphate-buffered saline, Life Tech No. 20012068) for 15 min washed twice for 5 min with PBS and permeabilized with 0.3% Triton X-100 (Sigma # T8787) in PBS for 10 min. After washing with 4% FCS in PBS, cells were incubated with blocking solution (2% FCS, 2% bovine serum albumin (BSA) (Sigma # A2153), 0,1% Tween 20 (Sigma # P9416) in PBS) for 45 min. Subsequently, cells were incubated with primary antibodies for 60 min or at 4 °C overnight, washed three times, incubated with secondary antibodies for 30 min and washed three times with 4% FCS in PBS. The following antibodies were used for immunohistochemistry: Rabbit a-ZO-1 (Invitrogen No. 617300, 1:125), Mouse a-acetylated tubulin (Sigma No. T6793, 1:2000), Rabbit a-ErbB1 (Novusbio No. NBP-1-51439, 1:200), Mouse a-MRP-2 (Santa Cruz No. SC-59608, 100 µg/ml, 1:10), Rabbit a-Glut-2 (Santa Cruz No. SC-9117, 200 µg/ml, 1:20), Mouse a-Ezrin (BD Transduction No. 610602, 1:50), Mouse a-ErbB2 (Thermo Scientific No. MS-229-P0, 200 µg/ml, 1:20), Rabbit a-Occludin (ThermoFisher No. 71-1500, 0.25 mg/ml, 1:100), Rabbit isotype (Life Tech No. 86199), Mouse isotype (Life Tech No. 86599), Goat isotype (Life Tech No. 02-6202), Goat a-Rabbit AlexaFluor 488 (Thermo Scientific, No. A11008, 1:250), Goat a-Rabbit AlexaFluor 555 (Life Tech, A21428, 1:250), Goat a-Mouse AlexaFluor 488 (Life Technologies, A11001, 1:250), Goat a-Mouse AlexaFluor 555 (Life Tech, A21422, 1:250), Goat a-Mouse AlexaFluor 647 (Life Tech, A-21236, 1:250), Donkey a-Rabbit AlexaFluor 647 (Life Tech, A-31573, 1:250). After nuclear stain (DraQ5, Abcam No. ab108410 or DAPI, H-1200, Vector Laboratories) cells were stored in PBS or Vectashield (H-1200, VectorLaboratories). All steps were performed at room temperature (RT). Cells were imaged with ImageXpress Micro XLS and Micro XLS-C HCI Systems (Molecular Devices, US) and SP5 laser point scanning confocal microscope (Leica).

### Compound exposure

Caco-2 cells in OrganoPlates and Transwells were exposed to staurosporine or aspirin and barrier integrity was measured. Staurosporine was tested separately on five experiments of Caco-2 tubes cultured in OrganoPlate at passages: 48, 51, 53, 54, and 59. Aspirin was tested separately on three experiments of Caco-2 tubes cultured in OrganoPlate at passages: 48, 51, and 56. Cells were exposed for 125 h for interval measurements and for 24 h for real-time measurements. Concentrations of aspirin were 0, 0.1, 0.33, 1.11, 3.67, 12.12, 40 mM (Sigma No. A5376), and concentrations of staurosporine were 0, 0.4, 1.1, 3.3, 10, 30, 90 µM (Sigma No. S4400). Aspirin was dissolved in medium. Staurosporine was dissolved in medium with 0.9% DMSO (Sigma, No. D8418) for 90 µM and 0.3% for the other concentrations. Staurosporine preparations for the shorter-term exposure also contained 9% tox medium for 90 μM staurosporine and 3% tox medium for the other concentrations (500 ml of MEMα (Sigma No. M4526) with 6.25 ml of L-glutamine (Sigma No. G7513), 6 ml Tox Supplement (Sigma No. MTOXRTSUP).

### Barrier integrity assay in OrganoPlate

Medium in the apical perfusion channel was replaced by medium containing 0.5 mg/ml FITC-dextran (150 kDa, Sigma No. 46946) and TRITC-dextran (4.4 kDa, Sigma No. T1037) and increasing concentrations of staurosporine or aspirin. Leakage of the fluorescent probe from the lumen of the tubular structure into the ECM compartment was automatically imaged using an ImageXpress XLS Micro HCI system at 37 °C and 5% CO2. For long-term exposure, imaging was performed at 1-h intervals up to 12 h, and from 24 to 36 h, and at 16, 35, 36, 48, 53, 60, 72, 82, 96, and 125 h. Between each interval, the OrganoPlate was placed back into the incubator on the interval rocker platform in order to maintain perfusion flow. For real-time measurement, automatic imaging was performed every hour for 24 h without removing the OrganoPlate from the HCI system. The ratio between the fluorescent signal in the basal and apical region of the tube was analyzed using FiJi^22^.

### Barrier integrity assay in Transwell

14–21 h prior to start of the experiment, media was changed to phenol-red free media (DMEM/F12 (Gibco, 11039-021), 10% FBS HI (Sigma, F4135), 1% NEAA (Life Tech, 11140050), 1% pen/strep (Sigma, P4333)). At the start of the compound exposure, medium was replaced. A total of 550 µl was added to the basolateral side, 250 µl of FITC-dextran solution (0.125 mg/ml medium) mixed with the compound of choice was added to the apical side. At each timepoint a 75 µl aliquot was collected from the basolateral side. 75 µl of fresh medium was added to the basolateral side after the 2 h aspirin timepoint. The fluorescence intensity was measured with a multi‐well plate fluorimeter (Fluoroskan Ascent FL, Thermo Fisher) with excitation at 485 nm and emission at 535 nm.

### Statistics and data analysis

Barrier integrity assay images were analyzed using FiJi^22^. Fluorescence intensities where measured in the apical and basal regions of the tubes and the ratio between these was reported. Tubes that reached a 40% fluorescent intensity ratio at first hour of measurement were considered non-leak tight and discarded. GraphPad Prism 6 (GraphPad Software Inc., La Jolla, CA) was used to generate Kaplan-Meier curves using the Survival Analysis – Survival Curve function defining an event as showing a basal to apical fluorescence intensity ratio over 40%. To prevent the overlapping data, curves were nudged by 1 data point each for clarity. Curve difference was estimated using log-rank (Mantel–Cox) test. Trend significance was evaluated using log-rank test.

Concentration-response curves were fitted using non-linear regression of the logarithm of the compound concentration vs. the normalized fluorescence assuming a top and bottom plateau at 0 and 100% and standard slope (hill slope = 1). The estimated EC_50_ values and 95% confidence interval were plotted vs. time.

### Data availability

The data that support the findings of this study are available from the corresponding author upon reasonable request.

## Electronic supplementary material

Supplementary Information
