# Supplementary material for: Membrane-free culture and real-time barrier integrity assessment of perfused intestinal epithelium tubes
Source: Nat Commun. 2017 Aug 15;8:262. doi: 10.1038/s41467-017-00259-3 (PMC5557798; doi:10.1038/s41467-017-00259-3)
Supplement: Supplementary file 1 — Supplementary Information [file 41467_2017_259_MOESM1_ESM.pdf]

File name: Supplementary Information
Description: Supplementary Figures

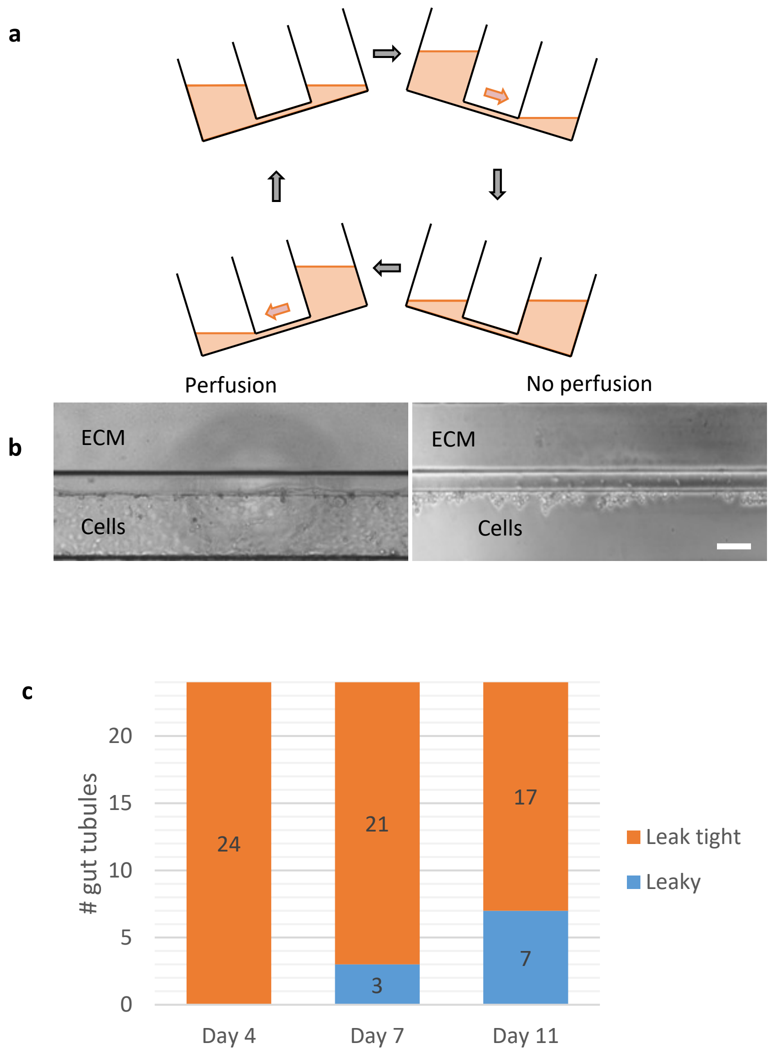

Supplementary Fig. 1 | Flow induction in OrganoPlate. (a) Flow is induced by leveling between two reservoirs that are connected by the perfusion channel. By placing the plate under an angle on an interval rocker that inverts the angle at regular intervals, a continuous bi-directional flow through the perfusion channel is induced. **(b)** Influence of flow on tube formation: A confluent tube of Caco-2 cells is formed within three days when perfused (left), while few cells survive culture without perfusion (right). Scale bars are 100µm. **(c)** Number of leak tight and leaky tubes in a single experimental run. Fluorescence intensity in the gel is measured and normalized to the fluorescence level in the tube channel. Upon crossing a threshold value of 0.4, a tube is considered leaky. At day 4 all tubes are leak tight, while at day 11 approximately 29% of tubes are leaking.

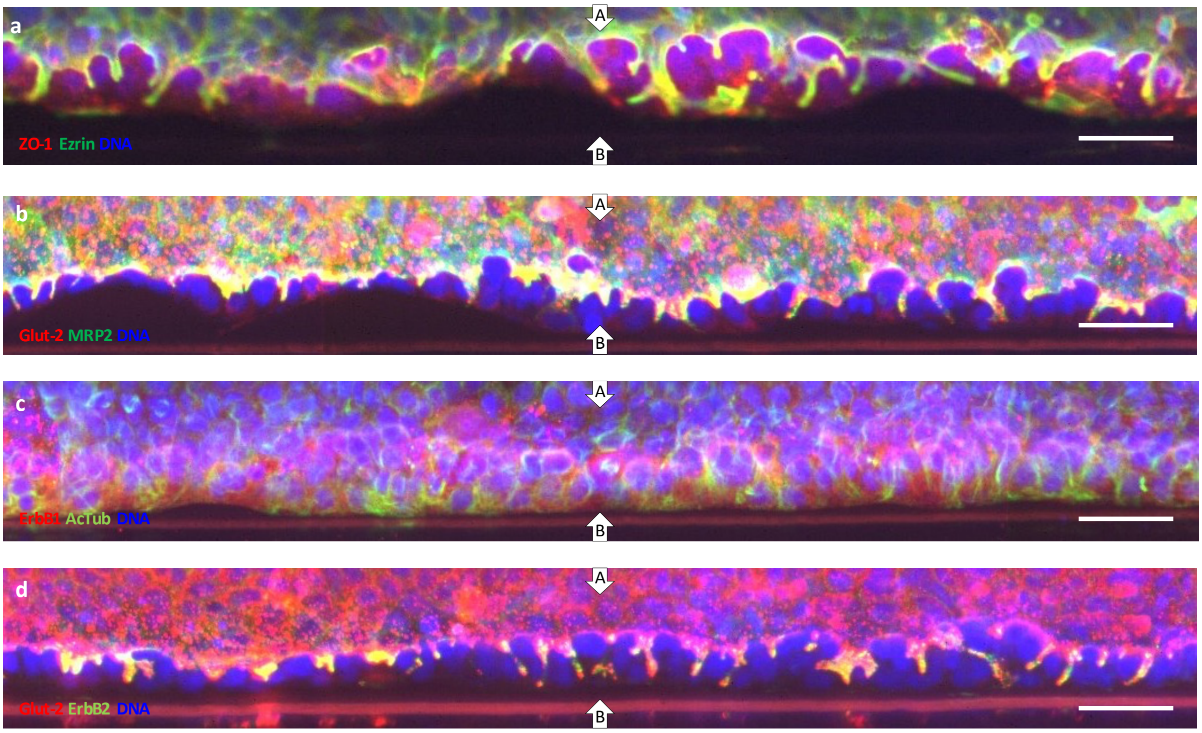

Supplementary Fig. 2 | Tubule characterization by immunofluorescent staining. Z-slices at higher magnification of confocal fluorescence micrographs of tubes from **fig. (2)** focusing on barrier morphology against ECM at approx. 50 µm above the bottom of the tube. An optical slice of the cells attached to the ECM is shown with white arrows indicating the apical (A) and basal (B) sides. The tube is stained for tight junctions (ZO-1 in red) and brush borders (Ezrin in green) showing apical positioning of Ezrin indicating polarization of the tube and invaginations expressing transport proteins. **(b)** expression of glucose and MRP2 transporters respectively stained with Glut-2 in red and MRP2 stain in green. Both stains clearly stain the apical side of the tube. **(c)** ErbB1 (red) and acetylated tubulin (green) expression. **(d)** Co-staining of Glut-2 transporter (red) and ErbB2 receptor (green); ErbB2 is primarily expressed pericellularly (here appearing as yellow). All tubes are fixated after four days in culture. Scale bars in white are 50µm. Images are representative of at least three biological and at least three technical replicates.

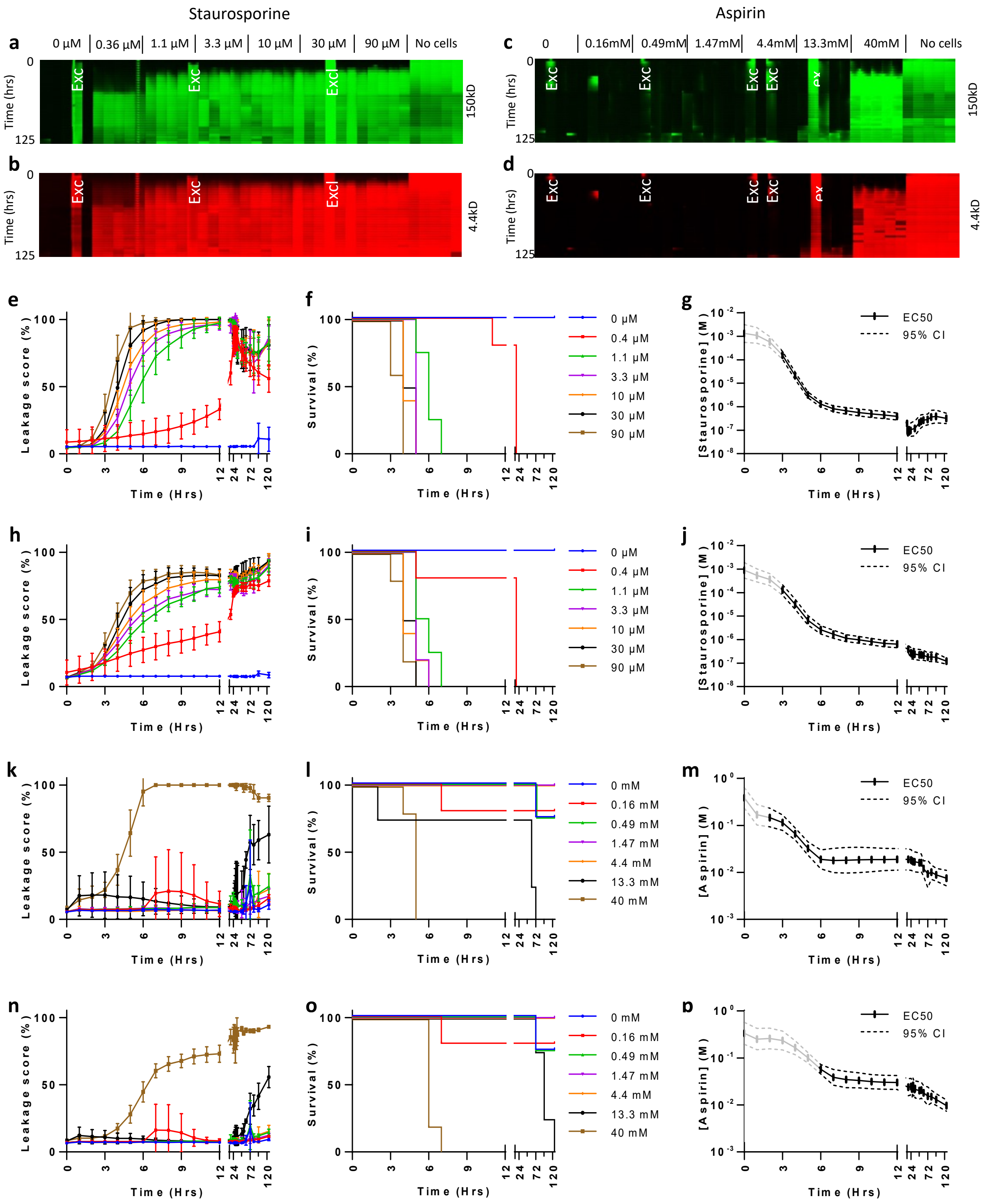

Supplementary Fig. 3 | Replicate of the experimental series in Figure 4 using cells at a different passage number, in which loss of barrier integrity is observed over time in a concentration-dependent manner for staurosporine (a, b, e-j) and aspirin (c, d, k-p). (a-d) Array of fluorescence micrographs of the gel region showing distribution of the 150kDa FITC-Dextran **(a, c)** and 4.4kDa TRITC-Dextran **(b, d)** over time and as a function of compound concentration; the loss of barrier integrity is shown by an increased fluorescent signal. Measurements are taken at 1-hour intervals up to 12 hours, at 16 hours, from 24 to 36 hours at 1-hour interval, and at 48, 53, 60, 72, 82, 96 and 125 hours. In between each interval, the OrganoPlate was placed back into the incubator on the interval rocker platform. Five technical

replicates of each concentration of a compound were measured on one single plate. Seven tubes were excluded from further data analysis, because the tubes appeared leaky at the first measurement (indicated with "Excl*") and one run was excluded because of a pipetting error (indicated with "Excl**"). **(e, h, k, n)** The progression of the loss of barrier function over time is plotted as the ratio between fluorescent signal in apical and basal regions for the various concentrations of staurosporine **(e, h)** and aspirin **(h, n)**; **(e, k)** show barrier integrity measurement using 150 kDa FITC dextran as a leakage marker, while **(h, n)** show barrier integrity measurement using 4.4 kDa TRITC-dextran as a leakage marker. The plotted line is the mean of 5 technical replicate exposures minus excluded datapoints and error bars depict the standard deviation **(f, i, l, o)** Kaplan-Meier curves were generated where survival was defined as showing a leakage score below 40%. Overlapping curves were shifted by 1% for clarity purposes. **(g, j, m, p)** EC50 values as a function of exposure time. EC50 values where obtained by fitting a concentration response curve at each time point based on non-linear regression of normalized leakage scores using standard slope. EC50 values obtained from time points before the first event in the Kaplan-Meier plot, as indicated by grayed out line, should be interpreted with caution as the curve fit could be dominated by noise rather than biological effect. Technical replicates represent tubes seeded on the same plate and exposed in the same experimental session. Further independent replicate series for both staurosporine and aspirin are compaired in **Supplementary Fig. 4.**

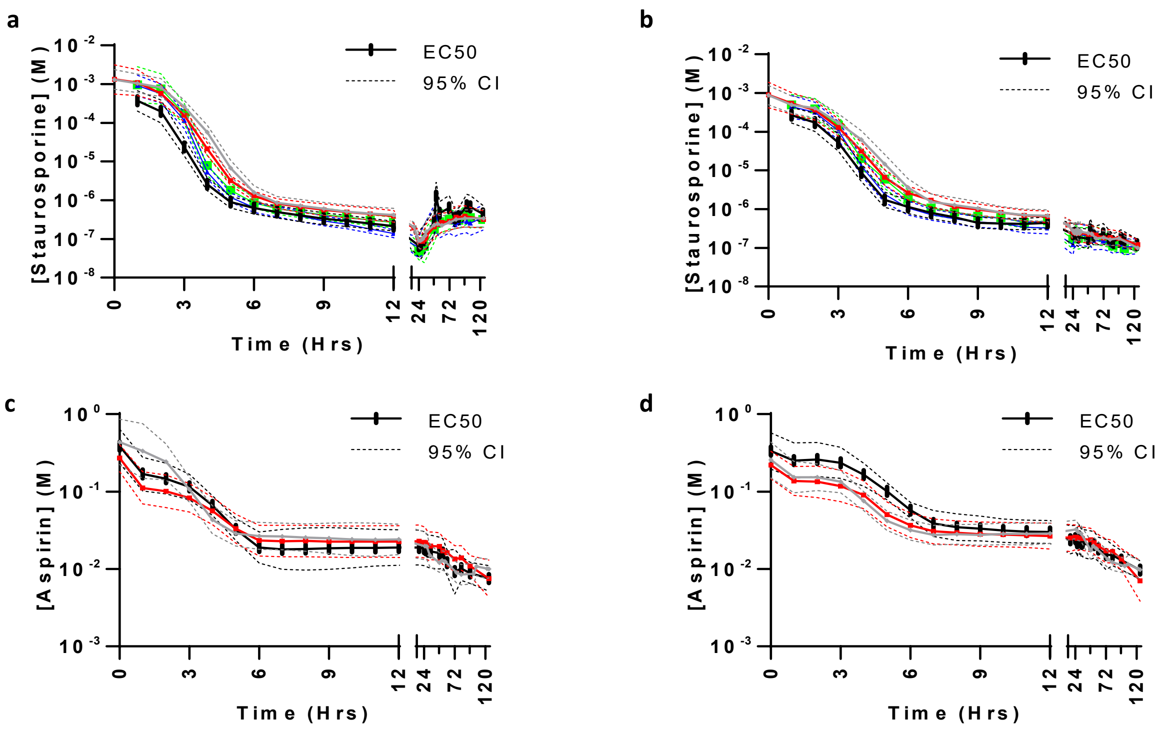

Supplementary Fig. 4 | Overlay of EC50 time curves of staurosporine and aspirin. The robustness of the assay was evaluated by comparing multiple replicate series of the experiments depicted in figure 4 and SI3 executed in separate experimental sessions using cells at different passage numbers. The EC50 time curves were generated for 5 independent staurosporine studies (**a, b**) and 3 independent aspirin studies (**c, d**). Figures **a, c** show results for 150kDa FITC-Dextran and figures **b, d** show results for 4.4kDa TRITC-Dextran. Independent experimental series show comparable results, confirming the robustness of the assays. The EC50 curves represent a total of 330 Caco-2 tubes and over 18,000 datapoints.

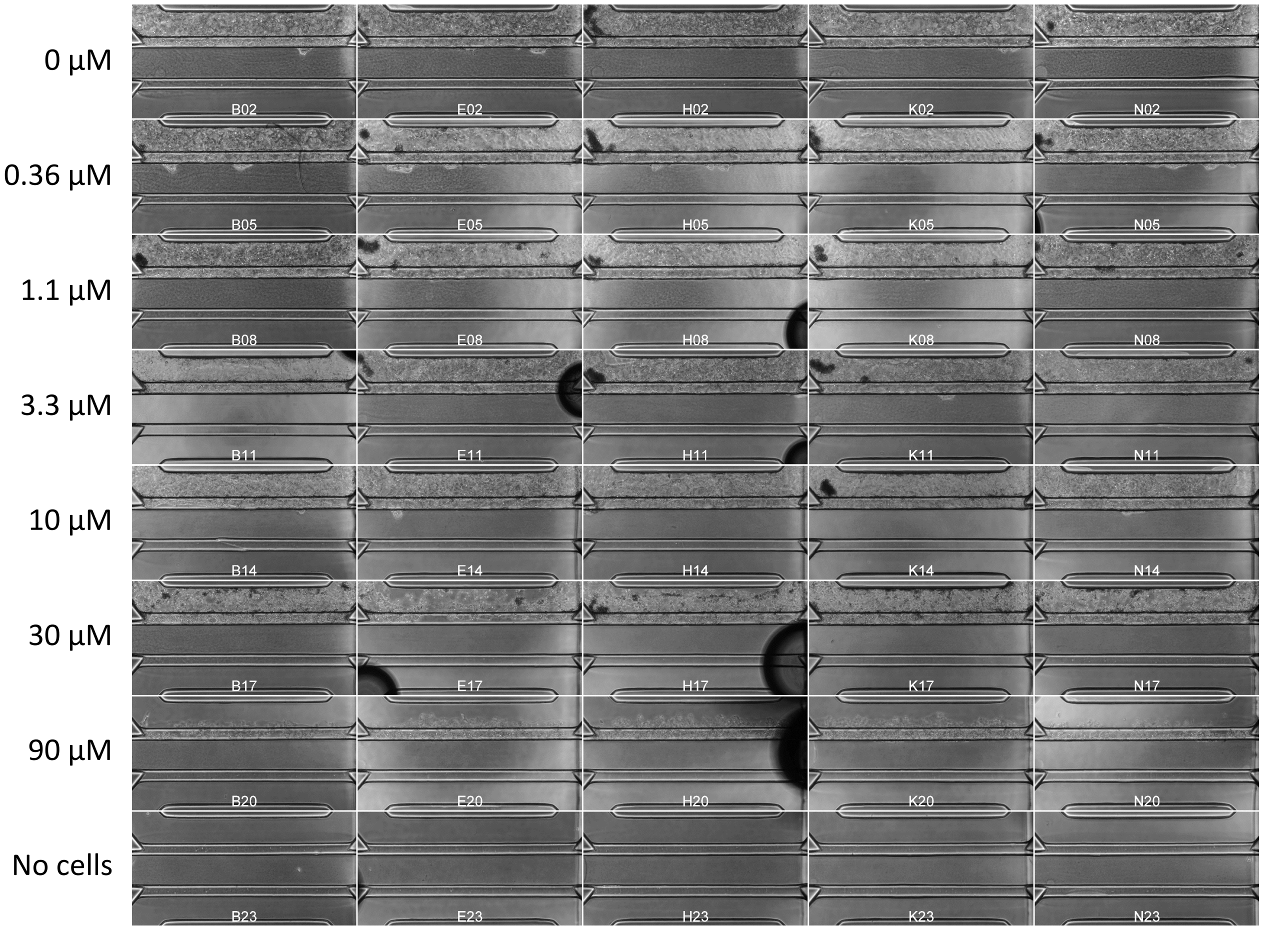

Supplementary Fig. 5 | Phase contrast images of Caco-2 tubes after 96 hours of staurosporine exposure. Even though all concentration already show leakage at this time point, a confluent monolayer is still observed at all but the highest concentration. At 90 µM tubes have fully deteriorated. Dead cells detach and are flushed away by the perfusion flow. Images are representative of 5 experimental series in quintuplicate.

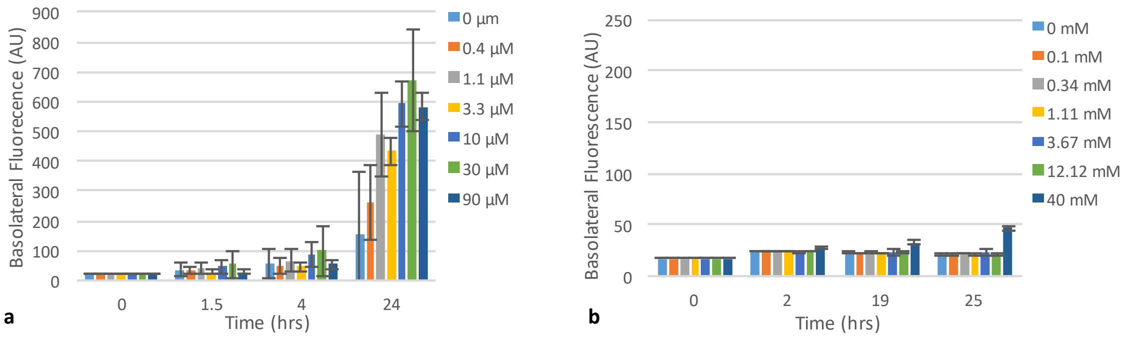

Supplementary Fig. 6 | Barrier integrity on Caco-layers in conventional Transwell systems exposed to (a) staurosporine and (b) aspirin. Caco-2 cells were seeded at a density of 60x10^3 cells/cm^2 on 6.5 mm polyester Transwell inserts with 0.4µm pores. Media was replaced three times per week (DMEM, 10% FCS, 1% NEAA, glutamax, p/s) for three weeks. After three weeks, the apical medium was replaced with 250 µL of medium containing a fluorescent probe (150kD FITC dextran) and staurosporine **(a)** or aspirin **(b)** at varying concentrations. The basolateral media was replaced with 550µL fresh medium. At various time points, 75µL basolateral medium was sampled to perform fluorescence measurements using a Fluoroskan FL plate reader. 75µL fresh medium was added after the aspirin 2hr timepoint to maintain sufficient sample for the remaining sampling time points. Fresh medium was used for 0 hour measurements. Four technical replicas were used for aspirin at 0 to 0.34 mM and three for the remaining concentrations. For staurosporine 4, 3, 3, 3, 4, 3, and 4 replicates were performed at 0, 0.4, 1.1, 3.3, 10, 30 and 90µM respectively.
